# Secondary Spontaneous Pneumothorax Caused by Pulmonary Schistosomiasis

**DOI:** 10.7759/cureus.18709

**Published:** 2021-10-12

**Authors:** Hanaa Bamefleh, Ghadah O Al-Hussain

**Affiliations:** 1 Anatomic Pathology, King Abdulaziz Medical City, Ministry of National Guard Health Affairs, Riyadh, SAU; 2 Pathology, King Abdulaziz Medical City, Ministry of National Guard Health Affairs, Riyadh, SAU

**Keywords:** bullae, pneumothorax, schistosoma mansoni, secondary spontaneous pneumothorax, pulmonary schistosomiasis, schistosomiasis

## Abstract

Schistosomiasis is an endemic disease commonly found in areas of Africa and the Middle East. It is a prevalent parasitic infection in the southern region of Saudi Arabia and Yemen. The most common species found are *Schistosoma mansoni *and *Schistosoma haematobium. *Schistosomiasis can manifest in the urinary bladder, liver, and gastrointestinal system. The occurrence of the infection in the lungs is very rare and usually appears after years of initial infection. We report a case of a 23-year-old Yemeni male who presented to the emergency department complaining of sudden, right-sided chest pain with shortness of breath of one day. Examination and chest X-ray revealed the presence of pneumothorax, and a chest tube was inserted accordingly. As the pneumothorax did not resolve and a continuous air leak was present, the patient was taken to the operation theatre on suspicion of a fistula. The diagnostic procedure found the presence of bullae and patterns of inflammatory infection. A resected lung wedge revealed the presence of Schistosoma eggs, and schistosomiasis was diagnosed. In conclusion, spontaneous pneumothorax secondary to infection can present in young healthy males. Meanwhile, schistosomiasis infection must be kept in mind when dealing with patients coming from endemic areas even if they present with no recent visit to endemic areas.

## Introduction

Schistosomiasis, also known as bilharziasis, is a parasitic tropical endemic disease prevalent in many areas of Africa and the Middle East [1]. It is estimated that there are around 700 million at risk of contracting the disease with 200 million active infections worldwide [1-2]. There are five species capable of infecting the human body: *Schistosoma mansoni*, *Schistosoma japonicum*, *Schistosoma mekongi*, *Schistosoma intercalatum*, and *Schistosoma haematobium* [3]. In Saudi Arabia, Schistosomiasis is one of the most common health problems, especially in the southern regions [2]. Furthermore, *Schistosoma mansoni* and *Schistosoma haematobium* are the two most common forms of Schistosomiasis in the country.

Clinically, Schistosomiasis can present within three to eight weeks of the infection or chronic, which is the most common presentation [2]. In the chronic phase, the eggs induce an immune reaction with granulomatous and fibrotic changes [4]. Moreover, the parasite can be latent for up to 40 years before it becomes active [5]. Schistosomiasis commonly affects the urinary and gastrointestinal tract causing a wide range of complications such as hematuria, bladder cancer, spontaneous abortion, ectopic pregnancy, and colon polyps [1,3]. However, its cardiopulmonary involvement is a rare event that occurs as a result of the involvement of the portosystemic collaterals or directly from the inferior vena cava [4]. If pulmonary involvement occurs, it can result in cor pulmonale due to the fibrotic changes that lead to pulmonary hypertension and in return cor pulmonale [4]. Clinically, fibrotic changes in the lung tend to prevent the lung from re-expanding making it susceptible to developing pneumothorax, hypoxia, and respiratory distress [6]. Histopathologically, the majority of pulmonary schistosomiasis are caused by *Schistosoma mansoni* with very few cases reporting *Schistosoma haematobium* and *Schistosoma japonicum* as the cause [4]. 

## Case presentation

**Figure 1:**
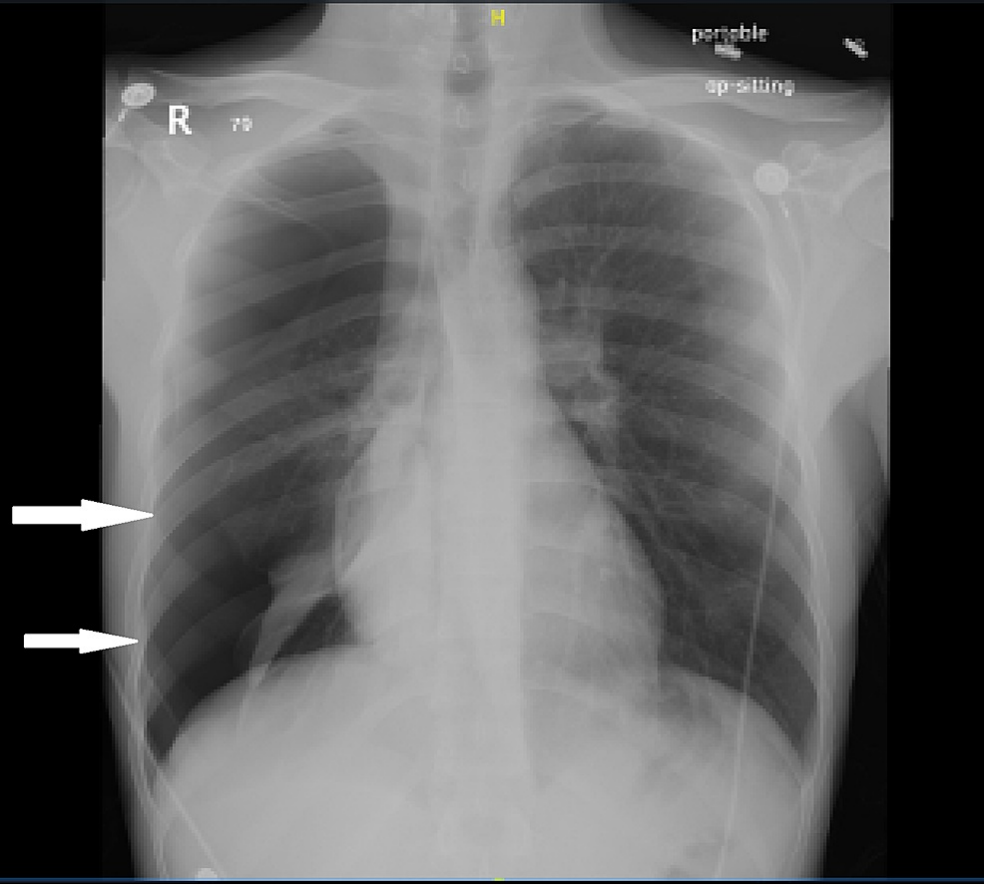
Chest X-ray showing collapsed lung and pneumothorax (white arrows)

A 24-year-old Yemeni male without any previous medical history presented to the emergency department complaining of sudden right-sided chest pain with shortness of breath after taking a shower earlier the same day. Upon questioning, there was no history of trauma, cough, recent travel to potentially endemic areas, recent infection, or any previous similar symptoms. Regarding his vital signs, he was afebrile with a heart rate of 78, respiratory rate of 20, blood pressure of 124/65 mmHg, and oxygen saturation of 96%. He was well-oriented and alert but showed signs of respiratory distress and difficulty in respiration. His chest examination revealed decreased air entry on the right side and normal breathing on the left side. Meanwhile, his other systemic examinations were unremarkable. His investigational panel included complete blood count (CBC) with differential was within normal limits except for isolated eosinophilia. Furthermore, electrolytes and cardiac markers were normal while tuberculosis acid bacilli were negative and blood culture showed no growth. Later, a chest X-ray (CXR) showed right-sided pneumothorax and a collapsed lung (Figure 1) with no mediastinal shift. Therefore, the patient was admitted, and a chest tube was inserted accordingly.

**Figure 2:**
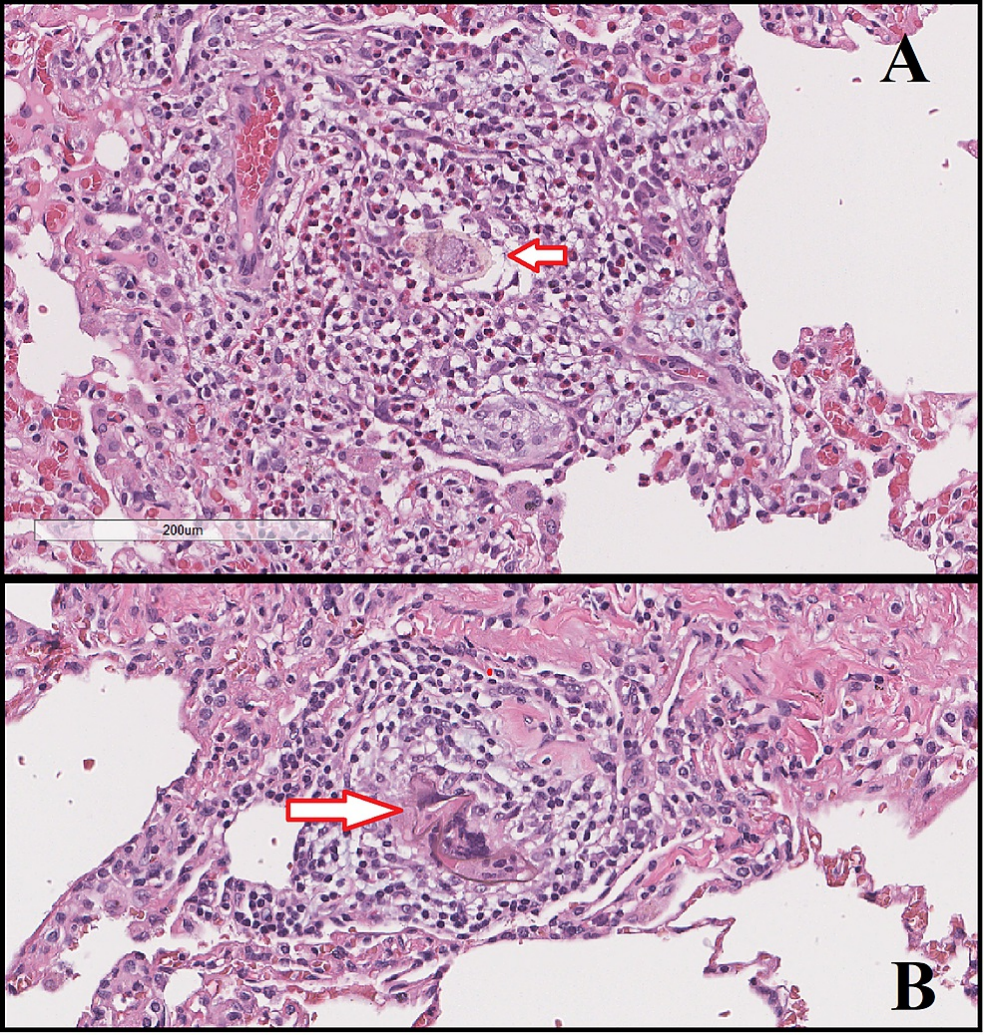
A) Hematoxylin and eosin (H&E) stain showing signs of schistosomiasis eggs of Schistosoma mansoni. B) Another section showing the same finding

The following day, a persistent air leak was noted in the chest tube without resolution of the pneumothorax, which raised the suspicion of the presence of a fistula. The following day, the patient underwent bullectomy, pleurectomy, and pleural abrasion via a video-assisted thoracoscopic surgery (VATS). The patient continuous leak was resolved, and there were no signs of an active leak in the following 24 hours. The resected wedge weighed 3 g and measured 3.5 x 2.0 x 1.0 cm. Histopathology cross-section of the specimen was stained with hematoxylin and eosin (H&E) stain showing signs of schistosomiasis eggs of Schistosoma mansoni (Figure 2) and was discharged on praziquantel with a follow-up appointment. The patient then failed to follow up further. 

## Discussion

Pulmonary schistosomiasis either be a chronic or acute infection is rarely diagnosed in its early stages [7]. Most often, the parasite and its eggs are found in the urinary bladder or the liver. In addition, incidences of gastrointestinal infection with schistosomiasis have been reported, which can cause ulceration and bleeding [3]. However, the incident of pulmonary infection is very rare and can lead to pulmonary hypertension. It is thought that the parasite can find its way to the liver first where the eggs can be sent through the circulation to the lungs. The obstruction and stress the eggs cause in the lungs lead to the development of portal-systemic collateral circulation leading to a hyperdynamic circulatory state. In return, this produces more stress on the pulmonary circulation with various immunological responses [7]. Furthermore, schistosomiasis is known for its vasculopathy effect on circulation. Therefore, the lungs arterioles undergo remodeling and hypertrophy, subsequently causing pulmonary hypertension [7]. In addition, the inflammatory process leads to the creation of granulomas. A study in Brazil on autopsies of 78 patients with hepatosplenic schistosomiasis found that 72% had periovular granulomas in the lung while remodeled pulmonary vessels were found in 46% of the patients [8].

Schistosoma haematobium has been linked to pulmonary manifestation and infection in previous reports [9-10]. The immunological changes in the lung often cause a diffused state of interstitial pulmonary fibrosis [4,7]. However, to the best of our knowledge, there was only one previously reported spontaneous pneumothorax caused by schistosomiasis in a 31-year-old man gold miner with evidence of urinary and liver schistosomiasis [10]. The changes in the pulmonary vasculature and the development of fibrosis can cause bulla formation and for air to be trapped similar to that found in emphysema [11]. Noppen reported in his review, that computed tomography in young children presenting with spontaneous pneumothorax had signs of bullae in the lungs [12].

Secondary spontaneous pneumothorax has been linked to various types of infections [12]. The most common presentation of secondary spontaneous pneumothorax is dyspnea, cyanosis, chest pain, and hypoxemia [12]. In our case, the patient presented initially with sudden chest pain and dyspnea. Curative therapy can promote the reversion of the vascular and inflammatory changes caused by the infection. However, such changes can persist or take up to 120 days to resolve [7]. Other studies showed that even after treatment and elimination of the parasite takes place, the reversal of the pulmonary remodeling takes place in acute infections only. Pulmonary remodeling caused by chronic infection of pulmonary schistosomiasis does not resolve and signs of clinical pulmonary hypertension persist [7]. 

## Conclusions

Pulmonary schistosomiasis is a very rare chronic infection that may cause secondary spontaneous pneumothorax. It can present years after a history of travel to endemic areas. If chest tube insertion does not resolve the lung collapse, surgical intervention is warranted to check for possible bullae and fistula formations that may be caused by the inflammatory and fibrotic changes caused in response to the parasitic infection.

## References

[1] Lackey EK, Horrall S (2020). Schistosomiasis (Schistosoma Haematobium). StatPearls.

[2] Baharoon S, Al-Jahdali H, Bamefleh H, Elkeir A, Yamani N (2011). Acute pulmonary schistosomiasis. J Glob Infect Dis.

[3] Akhtar MM, ALuhani N Sr, Younus D, ALahafi AH 4th, Abouhamda A (2020). Schistosomiasis mansoni manifesting as multiple colonic polyps. Cureus.

[4] Hajjar WM, Alsheikh AM, Alhumaid AY, Alkreedees NA, Abdulwahed NB, Hajjar AW (2018). Pulmonary schistosomiasis in a young male: a case report and review of the literature. Ann Thorac Med.

[5] Vieira P, Miranda HP, Cerqueira M (2007). Latent schistosomiasis in Portuguese soldiers. Mil Med.

[6] Porcel JM (2018). Secondary spontaneous pneumothorax in idiopathic pulmonary fibrosis: grim news. Respirology.

[7] Butrous G (2019). Schistosome infection and its effect on pulmonary circulation. Glob Cardiol Sci Pract.

[8] Andrade ZA, Andrade SG (1970). Pathogenesis of schistosomal pulmonary arteritis. Am J Trop Med Hyg.

[9] Doctor LR, Snider GL (1961). Diffuse interstitial pulmonary fibrosis associated with arthritis with comments on the definition of rheumatoid lung disease. Am Rev Respir Dis.

[10] Feldman C, Kallenbach J, Sutej P, Lewis M, Goldstein B (1986). Diffuse interstitial pulmonary fibrosis and spontaneous pneumothorax associated with Schistosoma haematobium infestation of the lungs. A case report. S Afr Med J.

[11] Kuhlman JE, Reyes BL, Hruban RH, Askin FB, Zerhouni EA, Fishman EK, Siegelman SS (1993). Abnormal air-filled spaces in the lung. Radiographics.

[12] Noppen M (2010). Spontaneous pneumothorax: epidemiology, pathophysiology and cause. Eur Respir Rev.

